# Effectiveness of Mind–Body Intervention for Inflammatory Conditions: Results from a 26-Week Randomized, Non-Blinded, Parallel-Group Trial

**DOI:** 10.3390/jcm10143107

**Published:** 2021-07-14

**Authors:** Thao Thi Nguyen, Christian G. Jensen, Lina Khoury, Bent Deleuran, Esther S. Blom, Thomas Breinholt, Robin Christensen, Lone Skov

**Affiliations:** 1 Unit of Social Medicine, Frederiksberg Hospital, 2000 Frederiksberg, Denmark; bich.Thao.thi.nguyen@regionh.dk; 2 Section for Biostatistics and Evidence-Based Research, The Parker Institute, Bispebjerg and Frederiksberg Hospital, 2000 Frederiksberg, Denmark; robin.christensen@regionh.dk; 3 Department of Dermatology and Allergy, Herlev and Gentofte Hospital, University of Copenhagen, 2900 Hellerup, Denmark; khoury_l@hotmail.com; 4 Department of Psychology, University of Copenhagen, 1165 Copenhagen, Denmark; cgj@fondenmentalsundhed.dk (C.G.J.); esorgenfrei@gmail.com (E.S.B.); 5 Department of Rheumatology, Aarhus University Hospital, 8200 Aarhus, Denmark; bd@biomed.au.dk; 6 TV 2 DANMARK A/S, Rugaardsvej 25, 5100 Odense, Denmark; tbre@tv2.dk; 7 Research Unit of Rheumatology, Department of Clinical Research, Odense University Hospital, University of Southern Denmark, 5000 Odense, Denmark

**Keywords:** biopsychosocial, mind–body intervention, treatment-as-usual, psoriasis, rheumatoid arthritis, health-related quality of life

## Abstract

Biopsychosocial intervention has been suggested as a complementary treatment strategy for patients with chronic conditions. We compared the effect of a mind–body intervention (MBI), relative to treatment-as-usual (TAU) on WHO-5 Well-being Index during an intensive period of 12 weeks and follow-up at week 26 among patients with either psoriasis (PsO) or rheumatoid arthritis (RA). The MBI was based on the ‘*Relaxation Response Resiliency Program*’ and the ‘*Open and Calm Program*’, as well as ‘*Mindfulness Based Stress Reduction*’ (MBSR). The trial was randomized, management-as-usual, and controlled. Statistical analyses were based on the intention-to-treat population using repeated measures and mixed effects models (NCT03888261). We screened 39 potential participants, 35 of which (PsO, *n* = 20; RA, *n* = 15) met the eligibility criteria and were randomized: 17 in the MBI group and 18 in the TAU group. Attrition from the intervention program was 19%, with 65% of MBI patients and 71% of TAU patients completing the outcome assessments. After 12 weeks, a statistically significant difference in WHO-5 was observed between the groups (*p* = 0.019). However, according to the protocol, during the entire trial period, the average (least squares mean values) WHO-5 score was higher although not statistically significant in the MBI group (65.3) compared with the TAU group (59.1), corresponding to a between-group difference over 26 weeks of 6.15 (95% CI: −0.26 to 12.56; *p* = 0.060). All things considered, adding biopsychosocial intervention to clinical practice to patients with conditions, such as PsO and RA, could potentially improve health-related quality of life.

## 1. Introduction

Chronic diseases are the most costly health conditions worldwide [1,2]. In addition, complex psychosocial factors such as depression, stress, work-related dynamics, and thinking patterns are thought to be associated with poor health status and impaired health-related quality of life (HR-QoL) among patients suffering from chronic inflammatory conditions such as psoriasis (PsO) and rheumatoid arthritis (RA) [3,4]. Currently, treatment of PsO and RA is primarily pharmacological with topical treatment being applied to mild cases of psoriasis and disease-modifying anti-rheumatic drugs (DMARDs), including biologics which are used to treat moderate-to-severe PsO and RA [5,6]. Considering the chronic nature of the diseases and the complex psychosocial factors of PsO and RA, nonpharmacologic therapies, such as biopsychosocial intervention, might be used as a complementary treatment strategy [7,8,9,10,11]. Indeed, several nonpharmacological non-invasive therapies, including mind–body interventions, have become available for chronic medical conditions in recent years [12,13,14,15]. Mind–body interventions are often part of the multidisciplinary rehabilitation and incorporate strategies that are thought to improve psychological and physical well-being, aiming to allow patients to take an active role in their treatment and promote people’s ability to cope with their condition and improve HR-QoL [14,16].

This pragmatic, randomized, and controlled trial was developed as a proof-of-concept study with the aim to explore the possible effectiveness of mind–body multidisciplinary rehabilitation on well-being and HR-QoL in patients with PsO and RA.

## 2. Materials and Methods

### 2.1. Trial Design and Registration

The trial was designed as a multicenter randomized, controlled, open-label, parallel-group trial conducted at two locations in Denmark over the course of 26 weeks. The trial was approved by the Regional Committees on Health Research Ethics and the Danish Data Protection Agency and was carried out in compliance with the Declaration of Helsinki and the International Conference on Harmonization Guidelines for Good Clinical Practice (H-18061530). The trial was registered before enrolling the first patient at the Clinical Trials Register (NCT03888261). Written informed consent was obtained from all participants before screening. All analyses were analyzed according to the intention-to-treat (ITT) principle, and rigorously followed the prespecified statistical analysis plan (available upon request).

### 2.2. Patients

Patients were recruited at routine visits from two specialist outpatient hospital clinics in Denmark: Gentofte University Hospital (Dermatology) and Aarhus University Hospital (Rheumatology), and from advertisements in local newspapers and patient associations. The eligible patients were seen from 15 January 2019 to 3 March 2020. In brief, patients eligible for inclusion were adults (aged ≥ 18 years) with a stable disease and no planned changes of therapy at the time of inclusion. For RA, the diagnosis had to be established according to American College of Rheumatology criteria [17] while the diagnosis of PsO was based on a clinical assessment. The following exclusion criteria were applied: (a) a lack of ability to participate due to physical and/or psychological reasons; (b) psychopathology: person with severe mental illness; (c) alcohol and/or drug abuse; (d) impaired cognitive functions unrelated to general distress; and (e) other immune-mediated conditions requiring systemic treatment. Patients fulfilling these eligibility criteria received written and oral information about the trial and were invited to a 1 h clinical assessment interview with a clinical psychologist to evaluate whether they were eligible and wished to participate. The patients were subsequently randomized to either the mind–body intervention (MBI) or treatment-as-usual (TAU) after completing the baseline measurements.

### 2.3. Randomisation, Allocation Concealment and Blinding

A computer-generated, permuted, and stratified randomization sequence was produced before any patients were enrolled and before participants were allocated in permuted blocks of 2 to 6 to the MBI or TAU group (1:1); the randomization sequence (stratified by center) was prepared by a biostatistician with no clinical involvement in the trial (R.C.). The allocation was concealed in a password-protected computer file only accessible by the biostatistician. Individual allocations were held in sealed, opaque, and consecutively numbered emails which were sent to and opened sequentially by one of the clinical psychologists (ESB) on site. SAS PROC PLAN was used to generate the randomization schedules, specifically SAS statistical software version 9.4 (SAS Institute Inc., Cary, NC, USA). For feasibility reasons, the open-label design was used since the personnel and patients involved in the intervention as well as patients in the control group immediately became aware of treatment allocation after randomization (opening the specific envelop) and were thus not blinded during the remaining trial period.

### 2.4. Mind–Body Intervention

The mind–body intervention (MBI) was developed for the purpose of the present trial based on the Open and Calm (OC) intervention [18], based on the relaxation response approach [19] and further developed into a meta-theoretical model proposing four common factor mechanisms of change (open attention, calm processing, conscious participation, and personal understanding) across several types of MBIs. OC thus explicitly recommends technical adaptations for contextually relevant purposes. The present MBI was thus structured according to the OC-protocol (a bodily-mental-social thematic cycle week-by-week) and focused on the OC core strategies, but also included body scans and hatha yoga exercises from mindfulness-based stress reduction (MBSR) [20] as a way of re-entering the body with gentleness, which is often challenging for patients suffering from physical ailments. The MBI was delivered by two clinical psychologists (CGJ and ESB) who were either trained in one of the interventions (CGJ) or both interventions (ESB). The intervention was group-based with a planned maximum of 15 participants per group who applied three therapeutic components:

1. Contemplative practices, including (a) exercises designed to activate a relaxation response in the body as well as body awareness (b) mindfulness meditation, and (c) zen-meditation.
2. Psychoeducation, i.e., educational and informative lectures, materials and pen-and-paper exercises concerning (a) physical, psychological, and social health promotion, (b) stress prevention and resilience, and (c) disease-specific mechanisms (e.g., typical symptoms, possible developmental paths, possible risks and beneficial effects, and specific health-related advice).
3. Dialogue, including therapist–group dialogue as well as participant–participant dialogue. Participants were paired in smaller units (2 participants per unit) and were encouraged to continue dialogues and discussion outside of the treatment sessions. Moreover, each participant was offered 5 individual consultations with the therapist.

The total duration of the intervention was 20–26 weeks, which was divided into a high-intensive and a low-intensive phase with the purposes of establishing and maintaining treatment effects, respectively. The high-intensive phase lasted 9 weeks with weekly 3 h group meetings. Additionally, the high-intensive phase included two individual consultations with the therapist in weeks 2–3 and in weeks 7–9. The first individual consultation focused on the motivation and the individual relevance and adjustment of what was being taught in the group sessions. The second individual consultation focused on summarizing experiences and individual strategies for sustaining and maintaining training and its consecutive effects.

The subsequent 12 weeks of the low-intensive phase comprised one 2–3 h monthly group meeting and one monthly individual consultation (of which 2 were delivered per patient on average). The group meetings were structured according to the biopsychosocial model, where each group meeting worked with mental health and mind–body techniques, mainly from either a physical, psychological, or social angle. Patients in the control group received TAU and participated in the scheduled assessments in week 12 and 24.

### 2.5. Patient-Reported Outcome Measures

The primary and key secondary outcomes of the current trial were based on patient-reported outcome measures using the World Health Organization Well-Being Index (WHO-5) and Medical Outcomes Study 36-Item Short-Form Health Survey (SF-36) [21,22]. The WHO-5 is a questionnaire consisting of 5 statements scoring from 0–5, yielding a raw score of 0–25, which is multiplied by 4 resulting in a total score of 0–100. A raw score below 13 or an answer of 0–1 in any of the five items indicate (risk of) low well-being [21]. The mean WHO-5 score in the general population was measured in different European countries. Thus, when using the WHO-5 as an outcome measure in clinical trials, the goal was often to reach the general population mean score; in Denmark the mean WHO-5 score was estimated to 70 [21]. The threshold for clinically relevant improvements is a change of at least 10 points on the WHO-5 [21,23]. The SF-36 is a multipurpose, short-form health survey with 36 questions [22] amounting to eight subscales divided across physical and psychological HR-QoL domains: physical function (PF), role physical (RP), bodily pain (BP), global health (GH), vitality (V), social function (SF), role emotional (RE), and mental health (MH). Subscale scores were combined to form our two secondary higher-order summary scores: the physical component summary (PCS) and mental component summary (MCS).

Outcomes were measured at baseline, at a 12-week visit (after the high-intensive period) and at the 26-week follow-up. Clinical assessments were carried out by the consultant specialist in the specific disease (psoriasis and rheumatoid arthritis, respectively). Because we expected maximal clinical effects of the combined intervention immediately after the intensive intervention (12 weeks), the primary outcome was chosen as the main group effect (across timepoints) from baseline to the week 26 visit in the WHO-5 Well-being Index.

The key secondary outcomes were the SF36 subscales: PCS and MCS from baseline to week 26. The exploratory secondary outcomes were changes in the eight individual SF36 subdomains.

### 2.6. Statistical Methods

As also stated in the SAP, the prespecified sample size was based on 30 participants with each of the conditions. It was previously estimated that a sample of 26 participants per randomized group would be required for the study to have 80% power to show a large clinical effect size (i.e., Cohen’s effect size of 0.8 related to the advantage of the Mind–Body Intervention over nothing) with respect to WHO-5, based on a two-sided type 1 error rate of 5%. Since it was anticipated that we would have some attrition during the week-26 follow-up, it was decided to aim for 60 participants in total, corresponding to approximately 30 individuals in each group. This would potentially correspond to a statistical power of 86% to detect a large clinical effect size (i.e., between the group comparisons, independent of condition). End points were analyzed according to the intention-to-treat (ITT) principle (whereby patients who stopped the assigned group intervention were invited for a follow-up), as stated in the statistical analysis plan (SAP; available upon request). The ITT principle asserts the effect of a treatment policy (that is, the planned treatment regimen), rather than the actual treatment given (i.e., independent of treatment adherence). To evaluate the longitudinal effects of the intervention, all outcome measures were analyzed using a multilevel repeated-measures linear mixed effects model (restricted maximum likelihood), with participant as random effects factor. The model included group (MBI vs. TAU), diagnosis (PsO and RA), and time point (0, 12, and 26 weeks from baseline) as fixed effects, also with the baseline value of the relevant variable as a covariate to reduce the random variation [24]. To assess the adequacy of the mixed effects linear models, we checked the validity of the assumptions made by reviewing both the systematic and the random parts of the models. We also investigated the model features via the predicted values and the residuals; that is, the residuals had to be normally distributed (around 0), independent of the predicted values (available upon request). Missing data were handled indirectly by statistical modelling using the repeated-measures linear mixed-effects models [25]. The primary contrasts between groups were estimated based on the main effect estimation (comparing the least squares means) from the repeated-measures analysis of covariance applied in mixed linear models (i.e., across 26 weeks of observations). While the primary analyses were based on all observed data that are valid under the plausible assumption that data are ‘*Missing at Random*’ (MAR) [26,27], we also performed sensitivity analyses to explore the effect of departures from the MAR assumption (e.g., a single-step, non-responder imputation using the value at baseline to replace missing data; these estimates will potentially be informative even if data are ‘*Missing Not At Random*’ [MNAR]).

All *p*-values and 95% confidence intervals (95% CIs) were two-sided. We did not apply adjustments for multiplicity, rather we analyzed the primary and key secondary outcomes in a prioritized order (i.e., “gatekeeping procedure”). The analyses of the secondary outcomes were performed in sequence until one of the analyses failed to show the statistically significant difference, or until all analyses were completed at the statistical significance level of 0.05 [25,28]. Cohen’s effect size (d) was used to assess and interpret the effect size of the applied interventions by comparing the groups after 12 weeks. The effect size was considered small (Cohen’s d = 0.2), medium (Cohen’s d = 0.5), or large (Cohen’s d = 0.8) [29].

In retrospect, after realizing the statistical power limitations related to the lower (than expected) number of patients (only 17 in each randomized group [34 participants in total]), we decided to explore what a new trial—based on the same assumptions—would correspond to in terms of statistical power. If a new trial was designed to detect a large clinical effect size (Cohen’s d = 0.80) with 34 patients enrolled (randomised 1:1), this would correspond to a statistical power of 62% which is clearly much lower than the recommended minimum of 80% statistical power when planning a new trial.

## 3. Results

### 3.1. Baseline Characteristics

**Figure 1:**
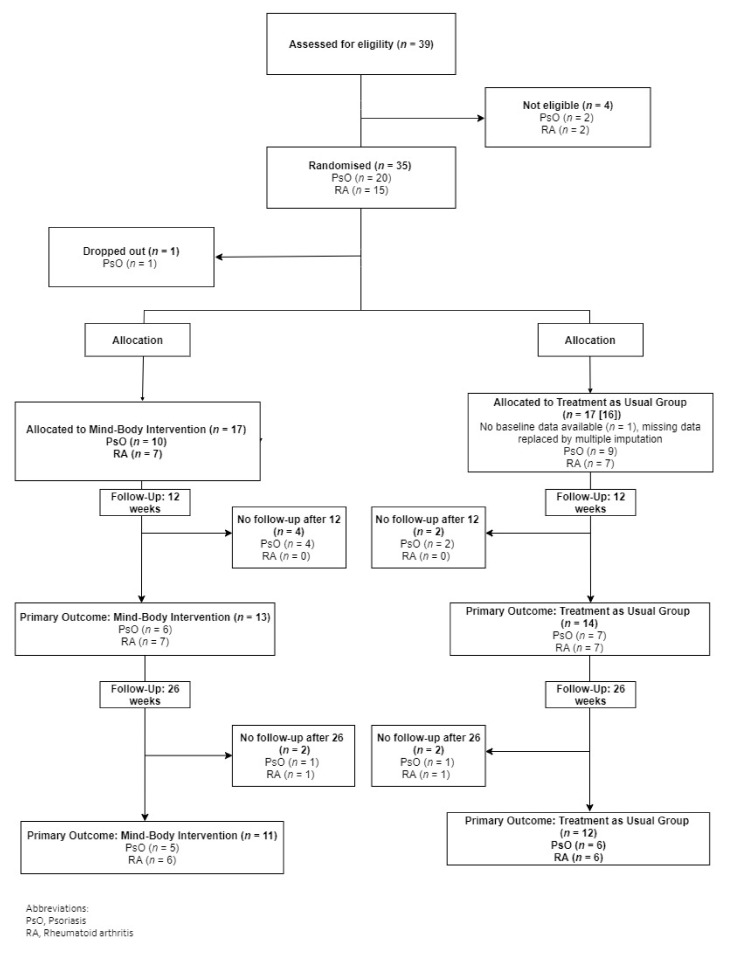
Trial Flow Diagram.

In total, 39 patients were assessed for eligibility. Four patients were excluded due to some of the listed exclusion criteria, leaving a trial of 35 patients who were included in the study (Figure 1). Of the included patients, 15 had RA and 20 had PsO, 10 of the patients in the MBI group had PsO and 7 had RA, whereas 10 had PsO and 8 had RA in the TAU group. One of the included patients with psoriasis dropped out following randomization but prior to having the baseline measurement assessed, and was consequently not part of the ITT population, resulting in 34 patients of which 17 (50%) were allocated to the MBI group and 17 (50%) in the TAU group. As presented in Table 1, the mean age of the ITT population was 48.0 (SD 12.7) years and 10 (29%) of the participants were males. The patients had a mean disease duration of 19.7 (SD 12.8) years, a mean entry weight of 80 kg, corresponding to an average body mass index (BMI) of 28 kg/m^2^. The mean baseline WHO-5 was 56.0 (SD 17.6) in the MBI group and 63.8 (13.5) in the TAU group. The baseline SF-36 MCS and PCS means were 49.7 (11.7) and 44.7 (9.9) in the MBI group and 50.8 (6.5) and 42.4 (10.0) in the TAU group, respectively.

### 3.2. Primary Outcome

**Figure 2:**
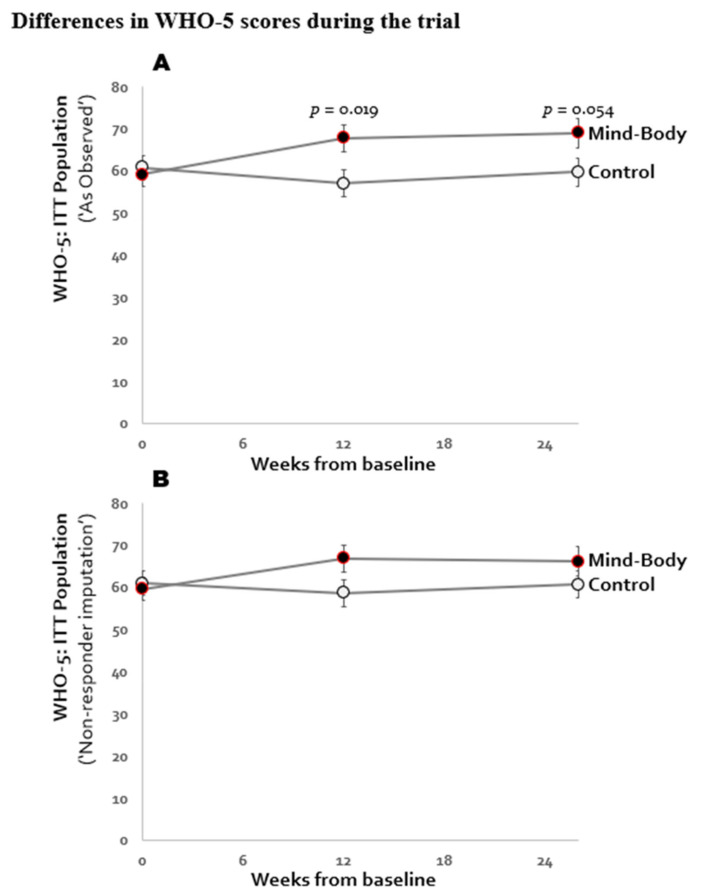
Least squared means WHO-5 from baseline throughout the 26-treatment period in the intention to treat (ITT) analysis, analyzing (**A**) as observed and (**B**) with non-responder imputation.

Eleven (65%) patients in the MBI group and twelve (71%) in the TAU group completed the 26-week measurement (Figure 1). The trajectory for the primary outcome, WHO-5 Well-being Index, from baseline to week 12 and week 26 is illustrated in Figure 2A. The test for interaction between group and time (3 levels) suggested a significantly different trajectory for the two groups: *p* = 0.035. Results of the primary analyses (across the entire 26-week period) are presented in Table 2: The MBI group showed a mean WHO-5 score of 65.3, whereas the TAU group showed a mean score of 59.1, amounting to a group difference of 6.15 points, approaching statistical significance (95% CI −0.26 to 12.56 points, *p* = 0.060). The results appeared robust to sensitivity analyses as illustrated in Figure 2B.

In contrast to the average effect (over all 26 weeks), after 12 weeks, there was a statistically significant difference between groups (67.8 vs. 57.0; difference between groups of 10.8 [95% CI 1.8 to 19.9]; *p* = 0.019). After the first 12-week trial period, the difference between groups potentially corresponded to a large effect size of Cohen’s d = 0.80. On the individual patient-level, the number of responders (individuals with an increase ≥10 WHO-5 units) were 7/17 (41%) of patients in the MBI and 2/17 (12%) in the TAU group. Nearly similar results were seen after 26 weeks, where 6/17 (35%) in the MBI group and 2/17 (12%) in the TAU group had maintained a clinically significant response (improved quality of life). MBI participants also qualitatively expressed high satisfaction with the program.

As presented in Table 2, an improvement in the PCS of the SF-36 of 3.05 [95% CI −6.25 to 0.16]; *p* = 0.062) points appeared to be present over 26 weeks in the intervention group when compared with the control group although this was not statistically significant. With regard to the mental component, there was no indication of an improvement in the SF-36 MCS of the SF-36 in either group during the 26-week period (Table 2).

Finally, according to the predefined statistical analysis plan, stratified analyses for the PsO and RA groups are descriptively presented separately in Supplementary Materials, Table S1 and S2; i.e., no formal statistical comparisons were carried out.

## 4. Discussion

In this pragmatic, clinical proof-of-concept, and randomized trial of patients with RA and PsO, the effectiveness of a new, biopsychosocial MBI was assessed. The result clearly indicated positive development of quality of life scores in the MBI group over time, and thus a trend towards better well-being measured by WHO-5 compared with usual clinical practice, although the primary statistical analysis (between group comparison across 26 weeks) was not statistically significant, appeared to be a potential effect immediately following intervention. In addition, a significantly higher proportion of patients achieved a clinically relevant change on the WHO-5 (≥10 points increase) in the MBI group compared with the TAU group.

Over the years, the efficacy of MBIs in different formats has been assessed in chronic somatic diseases [30,31,32,33,34]. A pilot study of 29 patients with severe psoriasis found an improved quality of life after 8 weeks of mindfulness-based cognitive therapy [35]. Contrary, a larger study comprising 72 participants with psoriasis found no improvement in quality of life and physical and psychological health compared with a control group receiving usual treatment after a 6 and 12 month follow-up, although participants reported that the mindfulness-based interventions were beneficial [36]. However, a recent review on psychological interventions for patients with psoriasis concluded that, due to limited transparency in reporting, low power, and lack of replications, nothing definitive could be concluded on these therapies [37]. Similarly, another review concluded that previous MBIs for psoriasis patients lacked methodological rigor and had largely failed to show psychological benefits [38]. In rheumatoid arthritis, an RCT comprising 72 patients found that patients receiving yoga-based mind–body intervention combined with DMARDs had greater reductions in depression symptoms, disease activity score-28, and erythrocyte sedimentation rate (DAS28ESR), compared with patients receiving only DMARDs [39]. Similarly, another study RCT comprising 57 patients with RA found yoga to be beneficial regarding depressive symptoms and fatigue, but not on HR-QoL compared with an educational control program [40]. In the current study, we observed a tendency towards a beneficial effect of MBI on well-being measured with WHO-5 and quality of life measured by the physical component of the SF-36 in patients with PsO or RA across 26 weeks, but the finding was not significant. Across the three time points, we observed a significantly more positive trajectory of well-being scores in the MBI group compared with the TAU group, and, immediately after the high-intensive phase (12 weeks), we observed significantly higher well-being scores in the MBI group compared with the control group receiving treatment as usual. However, we did not detect a significant group difference in the overall primary test of well-being across the three time points. This indicates that a more intensive follow-up with one or two group meetings a month for a period longer than 12 weeks may be needed for a more long-lasting improvement in well-being. This is well-known for other lifestyle changes such as weight loss, where group dynamics and close follow-ups are important to maintain a positive effect [41]. Overall, the patients included in our study had a mean WHO-5 at baseline of 59.9, which is considerably less than the mean WHO-5 in Denmark estimated at 70 [21]. A change in WHO-5 of ≥10 is considered clinically meaningful [21,23]. Here, we saw a markedly higher number of patients with a meaningful increase in WHO-5 at both 12 and 26 weeks in the MBI group compared to the TAU group.

Taken together, the results of the current study imply a positive effect of MBI on the well-being of patients with chronic diseases such as PsO and RA. However, the long-term effectiveness is still questionable and further research is urgently needed, including randomized trials with at least 50 patients in each group (i.e., more than 100 patients in total) such as the planned German SkinMind study [42]. Nevertheless, patients with psoriasis treated with biologics with complete remission still report impaired QoL [43]; complementary MBI might contribute to minimizing this. Moreover, a recent study showed that patients with psoriatic arthritis and psoriasis involving the hands, feet, genitals, or face were more likely to use MBI, thus the use of MBI might be a way to cope with the added mental component of visible psoriasis and the joint paint associated with psoriatic arthritis psoriasis involving the face, hands, feet or genitals and patients with psoriatic arthritis were more likely to use MBT, which could indicate MBT use to be used as a coping strategy for patients with visible and more severe disease [7].

Certain limitations should be considered when interpreting the result of the current study. First, the nature of the studied intervention prohibited a proper blinding of the patients. Thus, the apparent effectiveness might be due to a placebo response. Likewise, the primary investigator was unblinded to treatment allocation, which could have resulted in the observer bias ultimately leading to an overestimation of the effect on quality of life. Third, the planned study sample was 60 patients in total, but due to recruitment difficulties, only 35 were randomized with 34 patients in the ITT population, thus the failure to reject the null hypothesis might be due to a type II error. However, based on Cohen’s d-test, the effect size was found to be large after 12 weeks, suggesting a positive impact of MBI [29]. Strengths of the current study include the randomization of patients, thus potential confounders should not influence the results, the widely used and validated measures of well-being, and the inclusion of more than one chronic disease and center.

## 5. Conclusions

Management with mind–body biopsychosocial approaches in patients with inflammatory conditions such as psoriasis and rheumatoid arthritis could potentially improve health-related well-being if added to clinical practice. Despite the possible optimism bias implied here around the suspected clinical value related to MBI approaches in clinical practice, long-term effectiveness is still questionable and further research is urgently needed, including at least one confirmatory randomized trial with at least 50 patients in each group (i.e., more than 100 patients in total) in order to statistically detect a possible difference between groups of the magnitude we observed.

## Figures and Tables

**Table 1:** Baseline Characteristics and Values for Primary and Secondary Outcomes in the Combined ITT population.

Variable	Mind–Body Intervention Group (*n* = 17)		Treatment as Usual Group (*n* = 17)		Groups Combined (*n* = 34)	
Males, no (%)	5	29%	5	29%	10	29%
	**Mean**	**Std Dev**	**Mean**	**Std Dev**	**Mean**	**SD**
Age, years	51.7	11.7	44.4	12.9	48.0	12.7
Disease duration, years	19.1	14.8	20.4	10.8	19.7	12.8
Height, cm	170.2	16.5	169.5	9.5	169.8	13.3
Weight, kg	82.2	20.5	77.7	17.0	80.0	18.7
BMI, kg/m^2^	28.5	6.5	27.0	5.2	27.8	5.8
WHO-5, score: 0–100	56.0	17.6	63.8	13.5	59.9	16.0
SF-36 MCS, score: 0–100	49.7	11.7	50.8	6.5	50.2	9.4
SF-36 PCS, score: 0–100	44.7	9.9	42.4	10.0	43.6	9.9
SF-36 PF, score: 0–100	76.5	20.4	80.6	14.5	78.6	17.6
SF-36 RP, score: 0–100	67.6	39.3	59.0	42.1	63.3	40.4
SF-36 BP, score: 0–100	70.1	22.7	58.5	27.9	64.3	25.8
SF-36 GH, score: 0–100	51.8	18.5	50.7	19.7	51.3	18.8
SF-36 VT, score: 0–100	53.5	20.4	56.7	14.9	55.1	17.6
SF-36 SF, score: 0–100	80.3	25.8	77.4	19.9	78.8	22.7
SF-36 RE, score: 0–100	70.9	37.0	74.8	30.1	72.9	33.3
SF-36 MH, score: 0–100	78.2	14.8	77.4	9.9	77.8	12.4

SF-36 MCS, Medical Outcomes Study 36-Item Short-Form Health Survey, Mental Component Summary. SF-36 PF, Medical Outcomes Study 36-Item Short-Form Health Survey, Physical Function. SF-36 RP, Medical Outcomes Study 36-Item Short-Form Health Survey, Role Physical. SF-36 BP, Medical Outcomes Study 36-Item Short-Form Health Survey, Bodily Pain. SF-36 GH, Medical Outcomes Study 36-Item Short-Form Health Survey, Global Health. SF-36 VT, Medical Outcomes Study 36-Item Short-Form Health Survey, Vitality. SF-36 SF, Medical Outcomes Study 36-Item Short-Form Health Survey, Social Function. SF-36 RE, Medical Outcomes Study 36-Item Short-Form Health Survey, Role Emotional. SF-36 MH, Medical Outcomes Study 36-Item Short-Form Health Survey, Mental Health.

**Table 2:** Primary and key secondary outcomes during (across) the 26-week trial period.

	MBI (*n* = 17)		TAU (*n* = 17)		MBI vs. TAU			
Variable	LS Means	SE	LS Means	SE	Difference between Groups	LCL 95% to UCL 95%		*p*-Value
WHO-5, score: 0–100	65.3	2.2	59.1	2.2	−6.15	(−12.56 to 0.26)		0.060
SF-36 PCS, score: 0–100	45.1	1.1	42.1	1.1	−3.05	(−6.25 to 0.16)		0.062
SF-36 MCS, score: 0–100	51.3	1.0	50.6	1.0	−0.64	(−3.60 to 2.32)		n.e.
SF-36 PF, score: 0–100	81.6	2.5	74.3	2.5	−7.26	(−14.41 to −0.11)		n.e.
SF-36 RP, score: 0–100	71.9	4.6	60.6	4.6	−11.26	(−24.58 to 2.05)		n.e.
SF-36 BP, score: 0–100	69.4	2.9	62.3	2.9	−7.07	(−15.66 to 1.52)		n.e.
SF-36 GH, score: 0–100	56.2	2.0	52.0	2.0	−4.23	(−10.01 to 1.55)		n.e.
SF-36 VT, score: 0–100	58.6	2.8	49.5	2.7	−9.16	(−17.09 to −1.23)		n.e.
SF-36 SF, score: 0–100	81.4	2.7	79.2	2.7	−2.21	(−10.10 to 5.68)		n.e.
SF-36 RE, score: 0–100	80.01	3.7	76.9	3.7	−3.16	(−13.51 to 7.20)		n.e.
SF-36 MH, score: 0–100	79.0	1.6	77.5	1.6	−1.47	(−5.99 to 3.05)		n.e.

LsMeans, least-squares means. LCL95%, Lower Confident Limit. UCL95%, Upper Confident Limit. MBI, Mind–Body Intervention. TAU, Treatment as usual. WHO-5, The World Health Organisation—Five Well-Being Index. SF-36 PCS, MedicalOutcomes Study 36-Item Short-Form Health Survey, Physical Component Summary. SF-36 MCS, Medical Outcomes Study 36-Item Short-Form Health Survey, Mental Component Summary. SF-36 PF, Medical Outcomes Study 36-Item Short-Form Health Survey, Physical Function. SF-36 RP, Medical Outcomes Study 36-Item Short-Form Health Survey, Role Physical. SF-36 BP, Medical Outcomes Study 36-Item Short-Form Health Survey, Bodily Pain. SF-36 GH, Medical Outcomes Study 36-Item Short-Form Health Survey, Global Health. SF-36 VT, Medical Outcomes Study 36-Item Short-Form Health Survey, Vitality. SF-36 SF, Medical Outcomes Study 36-Item Short-Form Health Survey, Social Function. SF-36 RE, Medical Outcomes Study 36-Item Short-Form Health Survey, Role Emotional. SF-36 MH, Medical Outcomes Study 36-Item Short-Form Health Survey, Mental Health. n.e., not evaluated.

## Data Availability

The data presented in this study are available on request from the corresponding author.

## Supplementary Materials

The following are available online at https://www.mdpi.com/article/10.3390/jcm10143107/s1, Table S1: Values in each of the diagnoses: Psoriasis. Table S2: Values in each of the diagnoses: Rheumatoid arthritis.

## Abbreviations

WHO-5	The World Health Organisation-Five Well-Being Index
SF-36 PCS	Medical Outcomes Study 36-Item Short-Form Health Survey, Physical Component Summary
