# Supplementary material for: Effectiveness of Mind–Body Intervention for Inflammatory Conditions: Results from a 26-Week Randomized, Non-Blinded, Parallel-Group Trial
Source: J Clin Med. 2021 Jul 14;10(14):3107. doi: 10.3390/jcm10143107 (PMC8305779; doi:10.3390/jcm10143107)
Supplement: Supplementary file 1 [file jcm-10-03107-s001.zip › jcm-1252659 - supplemmentary.pdf]

*Article*

# Supplementary Material: Effectiveness of Mind–Body Intervention for Inflammatory Conditions: Results from a 26-Week Randomized, Non-Blinded, Parallel-Group Trial

**Thao Thi Nguyen, Christian G. Jensen, Lina Khoury, Bent Deleuran, Esther S. Blom, Thomas Breinholt, Robin Christensen and Lone Skov**

**Table S1.** Values in each of the diagnoses: Psoriasis.

| LsMeans (SE) | Treatment as Usual Group | | | Mind-Body Intervention | | |
|---|---|---|---|---|---|---|
| | **Week 0** | **Week 12** | **Week 26** | **Week 0** | **Week 12** | **Week 26** |
| WHO-5 | 60.3 (3.6) | 50.6 (4.5) | 51.9 (4.6) | 58.7 (3.6) | 67.1 (4.6) | 69.8 (5.0) |
| SF-36 PCS | 43.8 (1.9) | 43.6 (2.4) | 43.8 (2.4) | 44.0 (1.9) | 45.3 (2.7) | 42.4 (2.7) |
| SF-36 MCS | 50.2 (1.7) | 47.2 (2.2) | 45.7 (2.2) | 49.2 (1.7) | 50.2 (2.4) | 55.0 (2.4) |
| SF-36 PF | 78.9 (4.2) | 75.2 (5.4) | 72.2 (5.4) | 79.0 (2) | 79.8 (5.4) | 83.1 (5.4) |
| SF-36 RP | 64.5 (8.4) | 63.9 (10.9) | 59.9 (10.9) | 67.2 (8.5) | 73.1 (11.0) | 60.6 (11.0) |
| SF-36 BP | 62.0 (5.5) | 65.8 (7.1) | 72.3 (7.1) | 65.7 (5.5) | 69.8 (7.1) | 74.1 (7.1) |
| SF-36 GH | 52.0 (3.7) | 53.4 (4.7) | 46.3 (4.7) | 51.4 (3.7) | 56.8 (5.2) | 60.2 (5.2) |
| SF-36 VT | 55.6 (4.7) | 45.1 (6.1) | 46.8 (6.1) | 54.3 (4.7) | 53.9 (6.6) | 58.2 (6.6) |
| SF-36 SF | 80.6 (4.9) | 71.3 (6.3) | 74.9 (6.2) | 79.6 (4.8) | 79.8 (6.2) | 77.7 (6.2) |
| SF-36 RE | 76.7 (7.2) | 71.2 (9.3) | 56.7 (9.3) | 66.4 (7.3) | 78.3 (9.5) | 100.5 (9.5) |
| SF-36 MH | 77.7 (2.5) | 73.1 (3.2) | 70.1 (3.2) | 77.5 (2.5) | 77.6 (3.6) | 82.0 (3.6) |

**Table S2.** Values in each of the diagnoses: Rheumatoid arthritis.

| LsMeans (SE) | Treatment as Usual Group | | | Mind-Body Intervention | | |
|---|---|---|---|---|---|---|
| | **Week 0** | **Week 12** | **Week 26** | **Week 0** | **Week 12** | **Week 26** |
| WHO-5 | 60.2(4.2) | 62.4(4.2) | 67.0 (4.5) | 59.0(4.2) | 68.7 (4.2) | 68.5 (4.5) |
| SF-36 PCS | 43.5(2.2) | 42.5(2.2) | 35.5 (2.4) | 43.7(2.2) | 47.5 (2.2) | 46.7 (2.4) |
| SF-36 MCS | 48.3(2.0) | 55.0(2.0) | 55.7 (2.2) | 50.6(2.0) | 51.2 (2.0) | 52.3 (2.2) |
| SF-36 PF | 79.0(5.0) | 77.4 (5.0) | 63.0 (5.4) | 79.2(5.1) | 84.9 (5.1) | 83.4 (5.4) |
| SF-36 RP | 61.6(10.1) | 63.2(10.1) | 49.4 (11.0) | 62.9(10.1) | 91.4 (10.1) | 72.9 (10.9) |
| SF-36 BP | 61.9 (6.5) | 66.3 (6.5) | 45.9 (7.1) | 64.6 (6.5) | 71.4 (6.5) | 70.4 (7.1) |
| SF-36 GH | 51.0 (4.4) | 56.9 (4.4) | 51.7 (4.8) | 52.1 (4.4) | 56.3 (4.4) | 60.7 (4.7) |
| SF-36 VT | 54.4 (5.6) | 50.2 (5.6 | 43.6 (6.1) | 53.9 (5.6) | 65.5 (5.6) | 63.3 (6.1) |
| SF-36 SF | 75.0 (5.9) | 87.1 (5.9) | 83.0 (6.4) | 78.2 (5.8) | 85.0 (5.8) | 86.3 (6.2) |
| SF-36 RE | 68.7 (8.6) | 96.5 (8.6) | 86.4 (9.2) | 79.8 (8.7) | 76.7 (8.7) | 81.1 (9.4) |
| SF-36 MH | 77.6 (3.0) | 81.9 (3.0) | 83.2 (3.3) | 78.1 (3.1) | 78.4 (3.1) | 80.3 (3.3) |

Abbreviations: LsMeans, least-squares means; WHO-5, The World Health Organisation- Five Well-Being Index; SF-36 PCS, Medical Outcomes Study 36-Item Short-Form Health Survey, Physical Component Summary; SF-36 MCS, Medical Outcomes Study 36-Item Short-Form Health Survey, Mental Component Summary; SF-36 PF, Medical Outcomes Study 36-Item Short-Form Health Survey, Physical Function; SF-36 RP, Medical Outcomes Study 36-Item Short-Form Health Survey, Role Physical; SF-36 BP, Medical Outcomes Study 36-Item Short-Form Health Survey, Bodily Pain; SF-36 GH, Medical Outcomes Study 36-Item Short-Form Health Survey, Global Health; SF-36 VT, Medical Outcomes Study 36-Item Short-Form Health Survey, Vitality; SF-36 SF, Medical Outcomes Study 36-Item Short-Form Health Survey, Social Function; SF-36 RE, Medical Outcomes Study 36-Item Short-Form Health Survey, Role Emotional; SF-36 MH, Medical Outcomes Study 36-Item Short-Form Health Survey, Mental Health.
